# Supplementary figures and images for: Potential problems of removing one invasive species at a time: a meta-analysis of the interactions between invasive vertebrates and unexpected effects of removal programs
Source: PeerJ. 2016 Jun 2;4:e2029. doi: 10.7717/peerj.2029 (PMC4893336; doi:10.7717/peerj.2029)

## Supporting Information 2

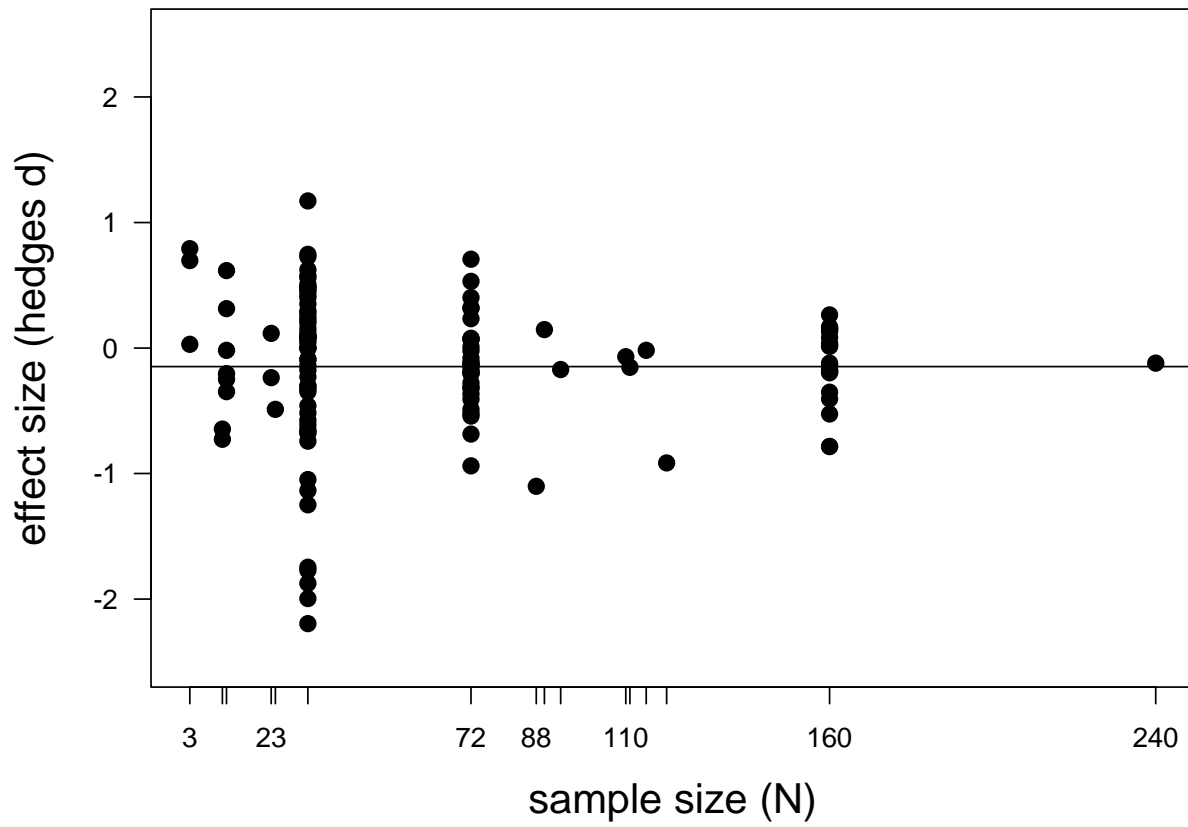

**Figure 4.** Funnel plot analysis of sample size against the Hedges' d value.

Supplement: Figure S1 [file peerj-04-2029-s002.pdf]
