# Supplementary material for: Potential problems of removing one invasive species at a time: a meta-analysis of the interactions between invasive vertebrates and unexpected effects of removal programs
Source: PeerJ. 2016 Jun 2;4:e2029. doi: 10.7717/peerj.2029 (PMC4893336; doi:10.7717/peerj.2029)
Supplement: Table S1 [file peerj-04-2029-s003.docx]

**Supporting Information 3**

**Table 3.** List of species and reference used for the analysis.

| **Species** | **Common name** | **Organism type** | **Foraging behavior** | **Native range*** | **Introduced range*** | **Reference** |
| --- | --- | --- | --- | --- | --- | --- |
|  |  |  |  |  |  |  |
| *Bufo marinus* | Cane toad | amphibian | carnivore | Southern Texas (USA), Mexico, Central America, northern South America and the Guianas, Trinidad and Tobago | Central America, southeastern USA, east Australia, China, Taiwan and Southeast Asia | Smith 2005 |
| *Osteopilus septentrionalis* | Cuban tree frog | amphibian | carnivore | Bahamas Cayman Islands, Cuba | Northern Florida, the Hawaiian island of Oahu, and throughout the Caribbean Islands | Smith 2005 |
| *Hemichromis bimaculatus*^1^ | African jewelfish | fish | carnivore | Western Africa, and from Algeria to Egypt | Florida, Hawaii (USA) | Porter-Whitaker et al. 2012 |
|  |  |  |  |  |  |  |
| *Salmo trutta* | Brown trout | fish | carnivore | Europe, northern Africa, and western Asia, including Iceland, Scandinavia, Russia, Corsica, Sardinia, and Sicily | Worldwide | Houde et al. 2015  Van Zwol et al. 2012 |
|  |  |  |  |  |  |  |
| *Trichosurus vulpecula* | Common Brushtail Possum | marsupial | herbivore | Australia and Tasmania | New Zealand | Didham et al. 2009 |
| *Cervus elaphus* | Red deer | mammal | herbivore | Eurasia and North America | South America, New Zealand, Australia, Ireland and Portugal | Wilson et al. 2006 |
| *Mus musculus* | House mouse | mammal | omnivore | Mediterranean region to China | Worldwide | Latorre et al. 2013  Wilson et al. 2006 |
|  |  |  |  |  |  |  |
| *Rattus rattus* | Black rat | mammal | omnivore | India, Pakistan | Worldwide | Latorre et al. 2013 |
|  |  |  |  |  |  |  |
| *Sus scrofa* | Wild boar | mammal | omnivore | Europe and Asia | Worldwide | Wilson et al. 2006 |
|  |  |  |  |  |  |  |
| *Oryctolagus cuniculus* | European rabbit | mammal | herbivore | Southwestern Europe (Spain and Portugal) and northwest Africa (Morocco and Algeria) | Worldwide | Latorre et al. 2013 |
| *Oncorhynchus kisutch*^2^ | Coho salmon | fish | carnivore | Central California to Alaska, through the Aleutian Islands, and from the Russia to Japan | Great Lakes (USA and Canada) | Houde et al. 2015 |
| *Oncorhynchus tshawytscha*^3^ | Chinook salmon | fish | carnivore | Pacific from Monterey Bay, California, Chukchi Sea, Alaska, and Siberia to Japan | Great Lakes (Canada and USA) and New Zealand | Houde et al. 2015 |
| *Cichlasoma urophthalmus* | Mayan cichlid | fish | carnivore | Central America, from Mexico to Nicaragua | Florida (USA), Singapore, Thailand | Porter-Whitaker et al. 2012 |
| *Oncorhynchus mykiss* | Rainbow trout | fish | carnivore | Eastern Pacific, from Alaska to Baja, California and Mexico | Worldwide | Houde et al. 2015  Van Zwol et al. 2012 |
|  |  |  |  |  |  |  |
|  |  |  |  |  |  |  |
| *Bos taurus* | Wild cattle | mammal | herbivore | Northern Africa, Europe, and Southern Asia | Worldwide | Didham et al. 2009 |
| *Oreochromis niloticus* | Nile tilapia | fish | omnivore | Africa from Egypt south to East and Central Africa, and as far west as Gambia, and Israel | Widely introduced, including Brazil, USA, Puerto Rico, Mexico | Oyugi et al. 2012 |
|  |  |  |  |  |  |  |
| *Cyprinus carpio* | Wild Common Carp | fish | herbivore | Europe, Siberia, China | Worldwide | Oyugi et al. 2012 |
|  |  |  |  |  |  |  |

* Sources (Date of access 07/04/2015):

http://www.iucnredlist.org/

http://animaldiversity.org/

http://nas.er.usgs.gov/

http://www.issg.org/database/species/.

^1^ Classified as invasive in INVASIVE DATABASE COMPENDIUM. CABI.

http://www.cabi.org/isc/datasheet/114748

^2^ Classified as invasive in Jonsson, B. (2011): NOBANIS – Invasive Alien Species Fact Sheet –

*Oncorhynchus mykiss* – From: Online Database of the European Network on Invasive Alien Species -

NOBANIS www.nobanis.org, Date of access 07/04/2015.

*^3^* Classified as invasive in MARINE INVASIVE SPECIES National Park Service

U.S. Department of the Interior

http://www.nature.nps.gov/water/marineinvasives/assets/PDFs/Oncorhynchus_tshawytscha.pdf
