# Supplementary material for: Potential problems of removing one invasive species at a time: a meta-analysis of the interactions between invasive vertebrates and unexpected effects of removal programs
Source: PeerJ. 2016 Jun 2;4:e2029. doi: 10.7717/peerj.2029 (PMC4893336; doi:10.7717/peerj.2029)
Supplement: Table S2 [file peerj-04-2029-s004.docx]

**Supporting Information 4**

**Table 4.** Extracted data used in meta-analysis.

| **Reference** | **Habitat type** | **Neighbor spp.** | **Focal spp. (excluded)** | **Native spp.** | **Response variable** |
| --- | --- | --- | --- | --- | --- |
|  |  |  |  |  |  |
|  |  |  |  |  |  |
| Didham et al. 2009 | temperate forest | *Bos taurus* (IH) | *Trichosurus vulpecula (IH)* | leaflitter invertebrate communities | native invertebrate density |
| Didham et al. 2009 | temperate forest | *Trichosurus vulpecula (IH)* | *Bos taurus* (IH) | leaflitter invertebrate communities | native invertebrate density |
| Houde et al. 2014 | seminatural freshwater stream tanks | *Salmo trutta* (IC); *Oncorhynchus mykiss* (IC); *O. tshawytscha* (IC); *O. kisutch* (IC) | *Oncorhynchus kisutch* (IC) | *Salmo salar* (NC) | condition; habitat use; length; mass; survival |
| Houde et al. 2015 | seminatural freshwater stream tanks | *Salmo trutta* (IC); *Oncorhynchus mykiss* (IC); *O. tshawytscha* (IC); *O. kisutch* (IC) | *Oncorhynchus mykiss* (IC) | *Salmo salar* (NC) | condition; habitat use; length; mass; survival |
| Houde et al. 2015 | seminatural freshwater stream tanks | *Salmo trutta* (IC); *Oncorhynchus mykiss* (IC); *O. tshawytscha* (IC); *O. kisutch* (IC) | *Oncorhynchus tshawytscha* (IC) | *Salmo salar* (NC) | condition; habitat use; length; mass; survival |
| Houde et al. 2015 | seminatural freshwater stream tanks | *Salmo trutta* (IC); *Oncorhynchus mykiss* (IC); *O. tshawytscha* (IC); *O. kisutch* (IC) | *Salmo trutta* (IC) | *Salmo salar* (NC) | condition; habitat use; length; mass; survival |
| Latorre et al. 2013 | Mediterranean garrigue | *Mus musculus* (IO) | *Rattus rattus* (IO) | *Medicago citrina* (NP) | seed predation |
| Latorre et al. 2013 | Mediterranean garrigue | *Rattus rattus* (IO) | *Oryctolagus cuniculus* (IH) | *Medicago citrina* (NP) | sapling predation; seedling predation |
| Oyugi et al. 2012 | artificial tanks aquaria | *Cyprinus carpio* (IH) | *Oreochromis niloticus* (IO) | artificial food | proportion of food taken |
| Porter-Whitaker et al. 2012 | wet prairie | *Cichlasoma urophthalmus* (IC) | *Hemichromis bimaculatus* (IC) | natural prey assemblage* | activity predator; vertical distribution; habitat use; predator-predator interaction; prey mortality rate |
| Porter-Whitaker et al. 2012 | wet prairie | *Cichlasoma urophthalmus* (IC) | *Hemichromis bimaculatus* (IC) | natural prey assemblage* | activity predator; vertical distribution; habitat use; predator-predator interaction; prey mortality rate |
| Smith 2005 | microcosms lab | *Bufo marinus* (IC) | *Osteopilus septentrionalis* (IC) | *Bufo terrestris*; *Hyla cinerea (NC)* | effect Gosner stage; metamorphosis effect; mass |
| Smith 2005 | microcosms lab | *Osteopilus septentrionalis* (IC) | *Bufo marinus* (IC) | *Bufo terrestris (NC)*; *Hyla cinerea (NC)* | effect Gosner stage; metamorphosis effect; mass |
| Van Zwol et al. 2012 | seminatural freshwater stream | *Oncorhynchus mykiss* (IC) | *Salmo trutta* (IC) | *Salmo salar* (NC) | aggressive acts; dominance; food items consumed; growth rate |
| Van Zwol et al. 2012 | seminatural freshwater stream | *Salmo trutta* (IC) | *Oncorhynchus mykiss* (IC) | *Salmo salar* (NC) | aggressive acts; dominance; food items consumed; growth rate |
| Wilson et al. 2006 | temperate forest | *Rattus spp.* (IO); *Mus musculus* (IO) | *Cervus elaphus*(IH); *Sus scrofa* (IO) | native woody seedlings (NP) | number of seedlings; composition of regenerating vegetation; relative ground cover |
|  |  |  |  |  |  |

*References:* (IC) invasive carnivore; (IH) invasive herbivore; (IO) invasive omnivore; (NC) native carnivore; (NP) native plant. * Natural prey assemblage in Everglades: *Palaemonetes paludosus*, *Gambusia holbrooki*, *Lucania goodei*, *Heterandria formosa* and *Jordanella floridae*.
