# Supplementary material for: Potential problems of removing one invasive species at a time: a meta-analysis of the interactions between invasive vertebrates and unexpected effects of removal programs
Source: PeerJ. 2016 Jun 2;4:e2029. doi: 10.7717/peerj.2029 (PMC4893336; doi:10.7717/peerj.2029)
Supplement: Table S3 [file peerj-04-2029-s005.docx]

**Supporting Information 5**

**Table 5.** Results from the reduced dataset* to test for issues of pseudoreplication.

|  | | **N** | **direction** | **Hedge’s D+** |
| --- | --- | --- | --- | --- |
| *HABITAT TYPE* | |  |  |  |
|  | forest | 3 | - | - 0.83 ± 0.26 |
|  | wetland | 6 | - | - 0.19 ± 0.13 |
|  | freshwater | 7 | - | - 0.18 ± 0.16 |
|  | garrigue | 1 | 0 | - 0.17 ± 0.28 |
| *EXPERIMENTAL DESIGN* | |  |  |  |
|  | experimental | 16 | - | - 0.17 ± 0.10 |
|  | experimental sample | 1 | - | - 1.10 ± 0.29 |
| *NATIVE RANGE OVERLAP* | |  |  |  |
|  | overlapping ranges | 7 | - | - 0.44 ± 0.19 |
|  | non-overlapping ranges | 10 | - | - 0.21 ± 0.11 |
| *INVASIVE FUNCTIONAL GROUP* | |  |  |  |
|  | amphibian | 4 | - | - 0.47 ± 0.26 |
|  | mammal | 4 | - | - 0.53 ± 0.19 |
|  | fish | 9 | - | - 0.13 ± 0.11 |
| *MANIPULATION* | |  |  |  |
|  | exclusion | 16 | - | - 0.17 ± 0.10 |
|  | exclusion neighbor | 1 | 0 | - 1.10 ± 0.29 |
| *TROPHIC POSITION OF REMOVED INVADER* | |  |  |  |
|  | carnivore | 12 | - | - 0.19 ± 0.10 |
|  | herbivore | 1 | 0 | - 0.03 ± 1.5 |
|  | omnivore | 4 | - | - 0.53 ± 0.19 |
|  |  |  |  |  |

* Houde et al. 2014 - survival; Van Zwol et al. 2012 -food items consumed; Smith 2005 - mass; Porter-Whitaker et al. 2012 - mortality rate; Oyugi et al. 2012 - food taken 3tilapia 1carp; Didham et al. 2009 - invertebrate density P0 F0 F12; Latorre et al. 2013 - predation seedling; Wilson et al. 2006 - relative ground cover.
