# Supplementary material for: Potential problems of removing one invasive species at a time: a meta-analysis of the interactions between invasive vertebrates and unexpected effects of removal programs
Source: PeerJ. 2016 Jun 2;4:e2029. doi: 10.7717/peerj.2029 (PMC4893336; doi:10.7717/peerj.2029)
Supplement: Supplemental Information 1 — Literature search: search terms use in a Web of Science® search for peer-reviewed studies on the impacts of removal a single invasive vertebrate species on native biodiversity. Details of the search conducted on the basis of genus of the terrestrial vertebrates invasive species reported among the 100 most damaging invasive species Global Invasive Species Database. [file peerj-04-2029-s001.docx]

**Supporting Information 1**

**Literature search**

Search terms use in a Web of Science^®^ search for peer-reviewed studies on the impacts of removal a single invasive vertebrate species on native biodiversity. Details of the search conducted on the basis of genus of the terrestrial vertebrates invasive species reported among the 100 most damaging invasive species Global Invasive Species Database

**Search:** (TS= (invas* OR introduced OR alien OR exotic OR nonnative OR "non-indigenous") AND TS=(interact* OR compet* OR facilit* OR meltdown) AND TS=(animal* OR tetrapod* OR amphib* OR eleutherodactylus OR gastrophryne OR lithobates OR rana OR litoria OR osteopilus OR rhinella OR bufo OR scinax OR xenopus OR reptil* OR anolis OR boa OR caiman OR chamaeleo OR ctenosaura OR elaphe OR gymnophthalmus OR hemidactylus OR iguana OR natrix OR norops OR podarcis OR phyton OR trachemys OR varanus OR bird* OR acridotheres OR alectoris OR anas OR branta OR bubo OR bubulcus OR carpodacus OR circus OR columba OR corvus OR cygnus OR estrilda OR gallus OR gymnorhina OR leiothrix OR molothrus OR myiopsitta OR oxyura OR passer OR pitangus OR porphyrio OR psittacula OR pycnonotus OR streptopelia OR sturnus OR zosterops OR mammal* OR marsupial* OR ammotragus OR axis OR bos OR bubalus OR callithrix OR camelus OR canis OR capra OR castor OR cavia OR cercopithecus OR cervus OR civettictis OR cricetomys OR crocidura OR cynictis OR equus OR erinaceus OR erythrocebus OR felis OR genetta OR hemitragus OR herpestes OR lama OR lepus OR lycalopex OR pseudalopex OR macaca OR martes OR mus OR mustela OR neovison OR myocastor OR nasua OR odocoileus OR oryctolagus OR ovis OR peromyscus OR petrogale OR phalanger OR procyon OR rangifer OR rattus OR rupicapra OR rusa OR sciurus OR suncus OR sus OR tamias OR tenrec OR trichosurus OR viverricula OR vulpes OR fish* OR clarias OR gambusia OR lates OR micropterus OR oncorhynchus OR oreochromis OR salmo) AND TS= animal* AND impact)) **Refined by document type:** (Article OR Review OR Proceedings Paper) AND **Web of Science**^®^ **Categories:** (Ecology OR Zoology OR Biodiversity Conservation OR Veterinary Sciences OR Multidisciplinary Sciences OR Forestry OR Environmental Sciences OR Ornithology OR Plant Sciences) AND **Research Areas:** (Environmental Sciences OR Ecology OR Zoology OR Biodiversity Conservation OR Veterinary Sciences OR Plant Sciences OR Forestry OR Veterinary Sciences OR Fisheries OR Water Resources OR Marine Freshwater Biology) **Timespan:** All years. **Index:}** SCI-EXPANDED, SSCI, A&HCI, CPCI-S, CPCI-SSH, BKCI-S, BKCI-SSH, CCR-EXPANDED, IC.
